# Comparing glaucoma risk in children receiving low‐dose and high‐dose glucocorticoid treatment after cataract surgery

**DOI:** 10.1111/aos.16746

**Published:** 2024-08-12

**Authors:** Diana Chabané Schmidt, Torben Martinussen, Ameenat Lola Solebo, Dorte Ancher Larsen, Daniella Bach‐Holm, Line Kessel

**Affiliations:** ^1^ Department of Ophthalmology Copenhagen University Hospital, Rigshospitalet Glostrup Denmark; ^2^ Department of Biostatistics University of Copenhagen Copenhagen Denmark; ^3^ Population, Policy and Practice Research and Teaching UCL Great Ormond Street Institute of Child Health London UK; ^4^ Great Ormond Street Hospital London UK; ^5^ Department of Ophthalmology Aarhus University Hospital Aarhus Denmark; ^6^ Department of Clinical Medicine University of Copenhagen Copenhagen Denmark

**Keywords:** congenital cataract, glaucoma, glucocorticoid, ocular hypertension, paediatric cataract, secondary glaucoma, steroid

## Abstract

**Purpose:**

Treatment with glucocorticoids following paediatric cataract surgery is crucial to prevent inflammation, but may lead to secondary glaucoma, and hypothalamic–pituitary–adrenal axis suppression. We wish to compare glaucoma outcomes following high‐dose and low‐dose glucocorticoid treatment after paediatric cataract surgery.

**Methods:**

This cohort study included Danish children undergoing cataract surgery before 10 years of age, receiving either a low‐dose or high‐dose postoperative glucocorticoid treatment. Case identification and collection of a standardized dataset were retrospective, from 1 January 2010 to 31 December 2016, and prospective thereafter, until 31 December 2021. High‐dose treatment included 0.5–1.0 mg subconjunctival depot dexamethasone or methylprednisolone, followed by 6–8 drops of dexamethasone for 1 week, tapered by one drop weekly. Low‐dose treatment included 6 drops for 3 days, followed by 3 drops for 18 days. Sustained (>3 months) ocular hypertension or glaucoma was compared between the two groups.

**Results:**

Overall, 267 children (388 eyes) were included in the study. Ninety‐five children (133 eyes) had received high‐dose treatment and had a median follow‐up time of 89 months (IQR: 57.2–107.4), while 173 children (255 eyes) had received the low‐dose treatment and had a median follow‐up time of 40.5 months (IQR: 22.9–60.4). Survival curves showed a lower risk of glaucoma in the low‐dose group for children with axial lengths ≥18 mm.

**Conclusion:**

Low‐dose glucocorticoid treatment was associated with a lower risk of glaucoma in children with axial lengths ≥18 mm. The same effect was not observed in children with shorter eyes. High‐dose glucocorticoid should be limited in children undergoing cataract surgery.

## 1 INTRODUCTION

Postoperative inflammatory control is crucial after paediatric cataract surgery, as inflammation can lead to the formation of synechiae, pupillary membranes and pupil‐block glaucoma (Whitman & Vanderveen, 2014). Glucocorticoid eye drops are used to reduce postoperative inflammation, but can increase intraocular pressure and subsequent sight‐threatening secondary glaucoma (Kaur et al., 2016). It is estimated that between 25% and 75% of children are steroid responders, defined as an intraocular pressure above 21 mmHg or an increase of greater than 5–10 mmHg compared to baseline (Feroze et al., 2023; Kaur et al., 2016; Kwok et al., 1997; Lam et al., 2005; Ohji et al., 1991). Additionally, glucocorticoid treatment can lead to the development of hypothalamic–pituitary–adrenal axis suppression, a condition where the endogenous production of cortisol is suppressed, potentially leading to a life‐threatening Addisonian crisis (Romano et al., 1977; Schmidt et al., 2023).

Thus, a balance between adequate inflammatory control and preventing glucocorticoid‐induced ocular hypertension (OHT) is important. There is no consensus on which postoperative treatment regimen after paediatric cataract surgery is optimal (Lambert & Shah, 2018; Self et al., 2020), but previous research suggests that a decrease in the dose and frequency of glucocorticoid treatment could result in fewer children with OHT and glaucoma (Lam et al., 2005; Ng et al., 2000).

Since 2010, all paediatric cataract surgery in Denmark has been performed at two specialized treatment centres: Rigshospitalet (RH) and Aarhus University Hospital (AUH). We have undertaken a multi‐site cohort study to examine the relationship between the dose of postoperative glucocorticoid treatment and the development of postoperative secondary glaucoma following surgery for paediatric cataract.

## 2 METHODS

The study included all children who underwent surgery for congenital, infantile or developmental cataract at one of the two specialized centres performing paediatric cataract surgery in Denmark, RH and AUH. Inclusion criteria comprised children who underwent cataract surgery before 10 years of age, between 1 January 2010 and 31 December 2021. Children undergoing surgery for secondary cataract caused by trauma, uveitis, steroid treatment, infections, cancer treatment, or those who had a diagnosis of OHT or glaucoma at the time of cataract surgery, were excluded from analysis. Cases were identified using hospital administrative databases.

### 2.1 Data collection

We collected data (using a standardized data collection form) on cataract aetiology, ocular and systemic diseases, age at time of diagnosis and cataract surgery, surgical approach and glucocorticoid treatment. Additionally, the age at time of diagnosis and treatment of glaucoma, OHT and visual axis opacity (VAO) were collected. Medical records from children who underwent cataract surgery from 1 January 2010 to 31 December 2016 were retrospectively reviewed, while children treated from 1 January 2017 to 31 December 2021 were followed prospectively.

### 2.2 Cataract surgery

Cataract surgery was performed by specialized surgeons and comprised either anterior or posterior approaches. The anterior approach included a limbocorneal incision, anterior capsulorhexis, lens aspiration combined with a rhexis of the posterior lens capsule, and an anterior vitrectomy. Posterior rhexis was omitted in older children (typically aged ≥7 years) deemed able to cooperate with neodymium:YAG (Nd:YAG) laser membranectomy treatment if necessary for subsequent VAO. Posterior approach comprised a pars plana incision, entry into the anterior chamber through the zonular fibres, anterior rhexis, lens aspiration, and lastly, anterior vitrectomy of part of the posterior capsule and anterior vitreous body. At AUH, intraocular lens (IOL) implantation was initially only performed on children >2 years of age. However, since 1 January 2017, the criteria were revised to include children >6 months of age. At RH, primary IOL implantation was performed on children >6 months of age during the whole study period. Children who underwent cataract surgery had follow‐up appointments with ophthalmologists on the first day, 1 week, and 1 month following the procedure. Thereafter they were monitored every 3 months until the age of 3 years, then every 6 months until the age of 7 years, and once a year until they turned 10 years.

### 2.3 Glucocorticoid treatment

Postoperative glucocorticoid treatment was categorized into a high‐dose and a low‐dose treatment. The high‐dose treatment comprised subconjunctival depot 0.5–1.0 mL methylprednisolone acetate 40 mg/mL or dexamethasone 4 mg/mL, instilled at the end of the surgery, and postoperative topical treatment with 6–8 eye drops of dexamethasone 1 mg/mL daily for the first week, tapering with a drop per week (Bangsgaard et al., 2018). In a few cases, the administration of subconjunctival glucocorticoid could not be verified by chart review in the high‐dose group. Until 2017, children at RH received the high‐dose treatment.

The low‐dose treatment consisted solely of topical treatment with six drops of dexamethasone 1 mg/mL daily for 3 days, followed by three drops daily for 18 days. The high‐dose treatment was approximately 2–4 times higher than the low‐dose treatment, considering methylprednisolone to be equipotent to 0.75 mg of dexamethasone (Bangsgaard et al., 2018; Liu et al., 2013; Schmidt et al., 2023). When the surgeon deemed it necessary, patients in the low‐dose group received 0.2 mL subconjunctival dexamethasone 4 mL/mg at the end of surgery, resulting in a cumulated glucocorticoid treatment of 4.4 mg dexamethasone. Compared to a standard high‐dose treatment consisting of approximately 9.7 mg dexamethasone, the low‐dose treatment, including depot dexamethasone, was substantially lower. The low‐dose treatment was the mainstay at AUH during the whole period and at RH from 2017.

### 2.4 Outcomes

The primary outcome was the incidence of glaucoma or OHT >3 months. The secondary outcome was the incidence of VAO.

Glaucoma was defined according to the guidelines from the World Glaucoma Association ninth consensus report (World Glaucoma Association & Weinreb, 2013) as at least two of the following criteria: Intraocular pressure above 21 mmHg, excavation of the optic nerve head, Haab striae or increased corneal diameter, myopic shift or an increase in axial length (AL) of the eye, or a glaucomatous visual field defect. OHT was defined as an ocular pressure >21 mmHg.

### 2.5 Statistics

**FIGURE 1:**
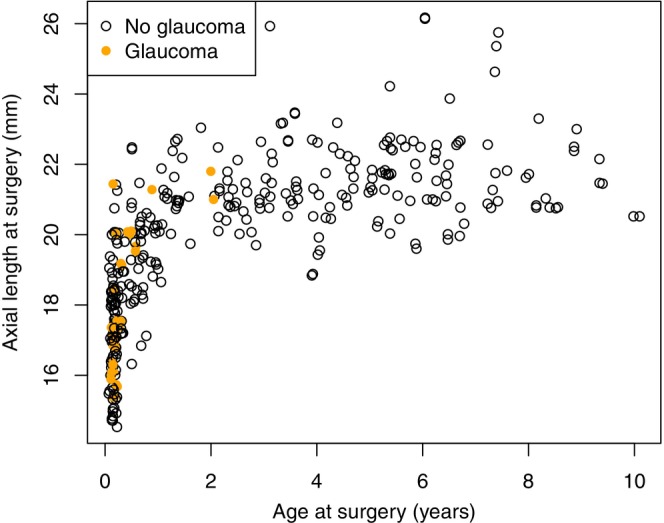
Scatter plot illustrating the axial length at the time of surgery compared to the age at cataract surgery in years. The orange dots are eyes diagnosed with glaucoma or ocular hypertension and the black eye are eyes not diagnosed with glaucoma or ocular hypertension at the latest follow‐up.

Average treatment effect (ATE) survival curves (Díaz et al., 2019) were used to compare the incidence of glaucoma between the low‐dose and the high‐dose group, using AL and IOL implantation as adjusting confounders, with 95% confidence intervals calculated considering paired data (eyes). Age at surgery was used as an adjusting confounder separately due to the high correlation with AL and IOL implantation. Age at surgery did not impact the treatment effect on the outcome, and the analysis was therefore focused on AL and IOL implantation as adjusting confounders. Aalens additive hazards model (Martinussen & Scheike, 2006) was used to judge the treatment effect in a multivariate regression setting, adjusting for the same variables as in the ATE‐analysis. Similar conclusions were obtained from the ATE survival curves and the multivariable regression analysis regarding the treatment effect. The use of robust standard errors had little impact on the results and was therefore not used in the subsequent analyses. The cut‐off value for axial length was determined by visually evaluating the scatter plot in Figure 1, choosing the value that most effectively distinguished between the eyes with or without glaucoma or OHT at the end of follow‐up. Standard Kaplan–Meier survival curves were used to determine the treatment effect on the incidence of treatment for VAO. Missing data caused by the lack of registration due to the partly retrospective design were excluded. Normality of data distribution was tested with Q–Q plots and variance with the Fligner‐Killeen test. In case of non‐normality but equal variance, the Mann–Whitney *U*‐test was used to compare groups. The statistical software R version 1.4.1717 was used (R Core Team, 2020).

### 2.6 Ethics statement

According to the Danish law on medical research ethics and a decision by the Medical Ethics Committee (Protocol no.: 16038234), an ethical board review was not required, as the study did not include additional patient examinations and data was collected in a patient treatment database. The study was approved by the Team for Medical Records Research, Centre for Health, Capital Region (Journal no.: R‐22046097), and the Danish Data Protection Agency (RH‐2016‐336; I‐Suite # 05070). The tenets of the Helsinki Declaration were followed.

## 3 RESULTS

We included 267 consecutive children (120 females) with 388 eyes undergoing paediatric cataract surgery. The median follow‐up time for the whole cohort was 51.9 months (IQR: 29.3–85.9). A genetic cause of cataract was found in 43 children (16%). Twenty‐four children (11%) had known syndromes or conditions, including Down syndrome (5), Nance Horan syndrome (3), Lowes syndrome (1), Glycogen Storage Disease (1), Warburg Micro syndrome (2), Stickler syndrome (2), Axenfeld‐Rieger syndrome (3), Klinefelter syndrome (1), Beckwith‐Wiedemann syndrome (1), Turner syndrome (1), cataract‐growth hormone deficiency‐sensory neuropathy‐sensorineural hearing loss‐skeletal dysplasia syndrome (CAGSSS) (1), Cerebrotendinous xanthomatosis (1) and Mannose‐binding lectin deficiency (2).

Ocular malformations were present in 43 eyes and included posterior embryotoxon (4 children, 5 eyes), iris coloboma (4 children, 5 eyes), lenticonus (2 eyes), microcornea (1 child, 2 eyes), microphthalmia (11 children, 16 eyes), persistent foetal vasculature (24 children, 27 eyes), sclerocornea and angle dysgenesis (1 eye), staphyloma (1 eye), and posterior synechiae (1 child, 2 eyes).

The age at cataract surgery was <6 months in 42% of children (41 children, 64 eyes) at AUH and in 30% (51 children, 76 eyes) at RH (Figure 1). The median follow‐up time at AUH and RH was 46.4 months (IQR: 21.7–68.4) and 46.8 months (IQR: 27.1–82.5), respectively. The median follow‐up time from cataract surgery was 40.5 months (IQR: 22.9–60.4) in the low‐dose group and 89.0 months (IQR: 57.2–107.4) in the high‐dose group.

There were fewer children with AL <18 mm than children with AL ≥18 mm at the time of surgery (Table 1). Eyes with AL <18 mm had a higher incidence of ocular malformations in the low‐dose (22.6%) and the high‐dose (28.6%) group compared to eyes with AL ≥18 mm in the low‐dose (9.4%) and the high‐dose (13.2%) group (Table 1). At the end of follow‐up, 12.5% of all eyes with microphthalmia had developed glaucoma or OHT.

**TABLE 1:** Descriptive data of children receiving low‐dose and high‐dose glucocorticoid treatment categorized according to axial length at the time of surgery.

	Low‐dose treatment^a^						High‐dose treatment^b^					
	All		AL <18 mm		AL ≥18 mm		All		AL <18 mm		AL ≥18 mm	
	Children	Eyes	Children	Eyes	Children	Eyes	Children	Eyes	Children	Eyes	Children	Eyes
Included children^d^	173	255	37	62	121	170	95	133	9	14	76	106
Female sex	78	112	18	27	54	77	42	59	4	6	31	45
Unilateral cataract		76		10		58		50		3		40
Persistent foetal vasculature	13	15	6	8	7	7	11	12	1	2	8	8
Ocular malformations	26	33	10	14	15	16	16	19	3	4	12	14
Primary IOL‐implantation	100	141	2	3	86	121	69	95	0	0	61	86
Subconjunctival depot glucocorticoid	5^c^	7	2	3	2	3	73	102	9	13	55	79

	Low‐dose treatment^a^			High‐dose treatment^b^		
	All	AL <18 mm	AL ≥18 mm	All	AL <18 mm	AL ≥18 mm
	Median (IQR)	Median (IQR)	Median (IQR)	Median (IQR)	Median (IQR)	Median (IQR)
AL at surgery (mm)	20.0 (17.6–21.5)	16.5 (15.8–17.3)	21.0 (19.7–21.9)	20.6 (19.5–21.3)	16.0 (15.5–16.7)	20.8 (20.0–21.4)
Age at surgery (days)	429 (70–1795)	61 (50–78)	1088 (215–2040)	577 (171–1670)	64 (54–72)	844 (234–1614)

Abbreviations: AL, Axial length; IOL, Intraocular lens.

^a^ Twenty‐three eyes were missing as the axial length was not registered.

^b^ Twelve eyes were missing as the axial length was not registered.

^c^ Five children received 0.2 mg depot dexamethasone treatment in the low‐dose group of which one was missing data on axial length.

^d^ One child had one eye treated with the low‐dose and one eye with the high‐dose treatment. Ocular malformations included embryotoxon, iris coloboma, lenticonus, microphthalmia, sclerocornea, staphyloma, posterior synechiae, microcornea, and persistent foetal vasculature.

### 3.1 Surgical intervention

The anterior approach had been used by seven specialized surgeons across both hospitals and the posterior approach by one surgeon. At primary cataract surgery, a higher proportion of children in the high‐dose group (71.4% of eyes) received an IOL compared to the low‐dose group (51.5% of eyes). Only one eye received a secondary IOL during the study period. The lens was implanted 6 years after primary cataract surgery and this eye did not develop glaucoma.

### 3.2 Glaucoma and ocular hypertension

Glaucoma or OHT had been diagnosed in 21 children (34 eyes) over the course of the observation period and in 13 children both eyes were affected. In all cases, the type of glaucoma was open‐angle. In eyes with glaucoma in the low‐dose group, IOP >21 mmHg was combined with an excavated papilla in seven eyes and an additional myopic shift in two of these eyes. In eyes with glaucoma in the high‐dose group, IOP >21 mmHg was combined with an excavated papilla in four eyes and an additional myopic shift in one of these eyes. Additionally, three eyes with glaucoma had IOP >21 mmHg and myopic shift. Four of the children with glaucoma or OHT (six eyes) had ocular malformations (microphthalmia, microcornea, microcornea combined with iris coloboma, posterior embryotoxon combined with rubeosis iridis and ectropion uveae, and irido‐lental synechiae) before the glaucoma or OHT diagnosis. Two children (three eyes) were born premature and one of them had cerebral palsy. Three children (five eyes) had cataract in relation to a syndrome (Nance‐Horan, Beckwith‐Wiedemanns syndrome, Lowe syndrome), and had all received the low‐dose treatment. One child (two eyes) had delayed development and dilated cardiomyopathy without a known cause. Fifteen children (25 eyes) had no ocular abnormalities and were healthy apart from the cataract. One child undergoing bilateral cataract surgery at the age of 6 months in the high‐dose group developed uveitis in one eye due to persistent lens material and was treated with glucocorticoids. Five years later, this eye developed glaucoma while the other eye was unaffected.

### 3.3 Glucocorticoid treatment and glaucoma incidence

**FIGURE 2:**
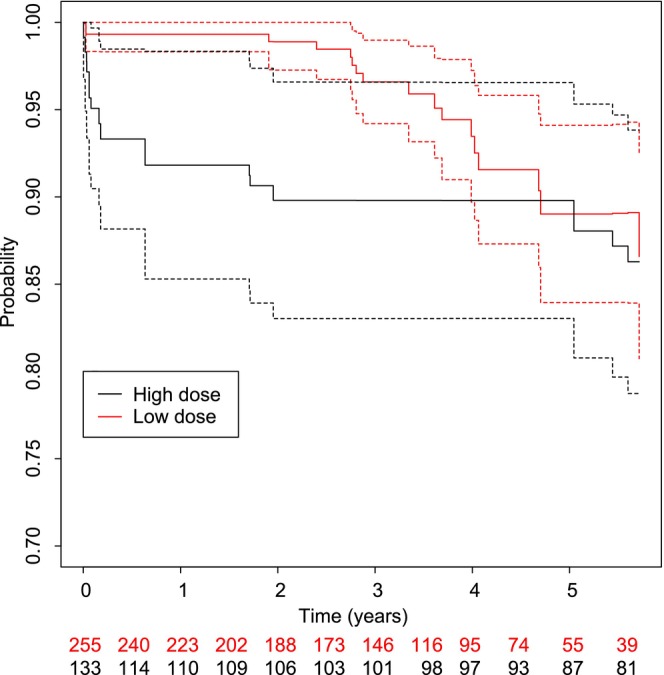
Average treatment effect survival curves concerning time to glaucoma or ocular hypertension after cataract surgery depending on the type of glucocorticoid treatment using axial length (< or ≥18 mm) and intraocular lens implantation (yes/no) as confounding factors. The timeline is years after cataract surgery and the dashed curves are 95%‐confidence curves. The number of children included in the analysis at a given time in both groups is illustrated at the bottom, red script is used for the number of children in the low‐dose group and black script for children in the high‐dose group.

Within the first 2 years after cataract surgery, the risk of glaucoma or OHT was estimated to be 10% (95% CI: 3.4–17) in the high‐dose group and 1% (95% CI: 0.0–2.7) in the low‐dose group. However, after 6 years there was no difference between the two treatment groups (Figure 2).

### 3.4 Visual axis opacification

**FIGURE 3:**
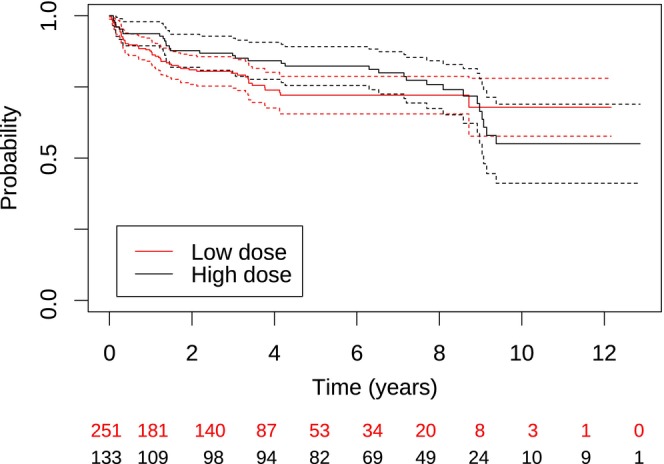
Kaplan–Meier curves depicting the risk of treatment (Nd:YAG laser membranectomy or vitrectomy) for visual axis opacification depending on the type of glucocorticoid treatment. The timeline is years after cataract surgery and the dashed curves are 95%‐confidence curves. The number of children included in the analysis at a given time in both groups is illustrated at the bottom.

During the observation period, 57 children (69 eyes) had been treated for VAO with either Nd:YAG laser or vitrectomy at least once. Children treated with Nd:YAG laser were older at the time of primary cataract surgery than children treated with vitrectomy, with a median of 6.5 years (IQR: 5.4–7.3) and 73 days (IQR: 51.8–205.5), respectively (*p* < 0.001, Mann–Whitney *U*‐test). There was no difference between the high‐dose and low‐dose group in the long‐term incidence of children requiring treatment for VAO (Figure 3).

### 3.5 Additional associated clinical factors

**FIGURE 4:**
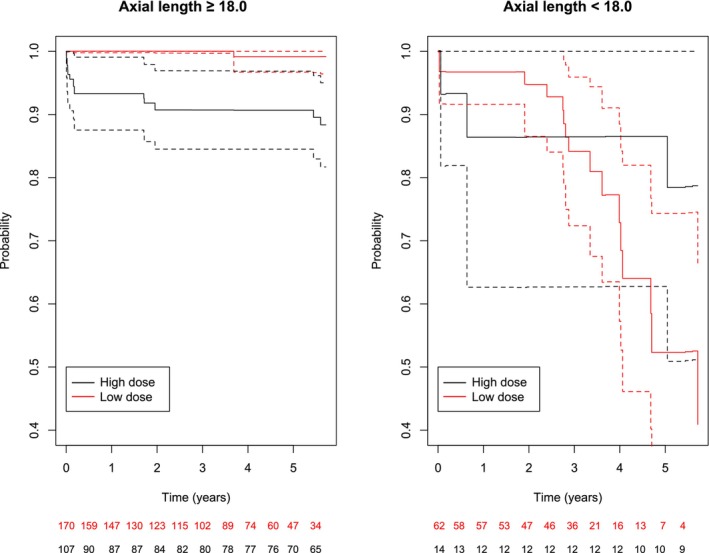
Average treatment effect survival curves concerning time to glaucoma or ocular hypertension after cataract surgery depending on the type of glucocorticoid treatment in children with axial lengths ≥18 and <18 mm using intraocular lens implantation (yes/no) as a confounding factor. The timeline is years after cataract surgery and the dashed curves are 95%‐confidence curves. The number of children included in the analysis at a given time in both groups is illustrated at the bottom.

Children with AL ≥18 mm at the time of surgery had a reduced risk of glaucoma or OHT when receiving the low‐dose treatment compared to the high‐dose treatment (Figure 4, left panel). This was also found in a multiple additive hazards regression analysis (adjusting for IOL implantation) (*p* = 0.008). The same effect of the low‐dose treatment was not found for eyes with AL <18 mm at the time of surgery (Figure 4, right panel).

Using age instead of AL, we found that children had a higher risk of glaucoma or OHT if they were <6 months of age at the time of cataract surgery. However, there was no significant interaction between age and treatment in the multiple additive hazards regression analysis (*p* = 0.43) in contrast to what we found for AL and treatment (Figure 4).

## 4 DISCUSSION

In our initial analysis on the incidence of glaucoma or OHT, there was no difference between the low‐dose and high‐dose glucocorticoid treatment after 6 years. However, when we stratified by AL < or ≥18 mm and adjusted for IOL implantation, we found a difference between the two treatment regimens. After 5 years, eyes with AL ≥18 mm receiving the low‐dose treatment had a lower risk of secondary glaucoma or OHT compared to the high‐dose treatment. Conversely, there was no effect of the glucocorticoid dose in eyes with AL <18 mm. The reason for the lack of difference in the secondary glaucoma risk 6 years after surgery between the low‐dose and high‐dose glucocorticoid treatment was driven by the eyes with AL <18 mm.

The increased number of children with AL <18 mm developing glaucoma or OHT could be attributed to younger age at surgery, a higher incidence of ocular anomalies, and eye maturity, possibly affecting the trabecular meshwork, which regulates the aqueous outflow (Remé & Lalive d'Epinay, 1981). Alternatively, eyes with smaller dimensions or malformations could be more susceptible to the microtubular alterations caused by dexamethasone, regardless of the treatment dose. However, we might not be able to detect any difference in the risk of glaucoma between the two treatment regimens, due to our relatively small cohort size. We found that children who had surgery <6 months of age had a higher risk of glaucoma or OHT regardless of treatment regime. As younger children have a stronger inflammatory response, they could need a higher glucocorticoid dose.

Other studies have also found an association between the development of glaucoma and younger age at surgery (Freedman et al., 2015; Haargaard et al., 2008; Solebo & Rahi, 2020). In line with our findings, the IOLunder2 study found that increasing age at the time of surgery and AL lowered the risk of glaucoma. Also 40% of the children in the IOLunder2 study received an intensive postoperative glucocorticoid treatment in the first week, consisting of eye drops every 2 h during daytime, followed by 4 times daily for 4 weeks, which resembles the high‐dose treatment regimen in our study (Solebo & Rahi, 2020).

Previous research suggested that children with aphakia have a higher risk of glaucoma (Asrani et al., 2000; Trivedi et al., 2006). This has not been confirmed by the 10‐year results of the Infant Aphakia Study or the 5‐year results from the IOLunder2 study (Freedman et al., 2021; Lola Solebo et al., 2018). As children in Denmark received IOL implantation >6 months of age at RH and at AUH after 2017, the variables age at surgery and IOL implantation are highly correlated.

We did not find an increased long‐term risk of treatment for VAO in the low‐dose group, suggesting that the low glucocorticoid dose was sufficient to prevent inflammation. However, the risk of treatment for VAO was higher in the first 5 years in the low‐dose treatment. This could lead to a higher rate of vitrectomies requiring anaesthesia, as younger children are unable to cooperate to an Nd:YAG laser membranectomy.

The limitations of this study are the non‐interventional design. Selection bias and unknown confounders may have had a negative impact on the internal and external validity of our study. The age at surgery varied from children only a few weeks old to school‐age children, as we included both congenital and developing cataracts. As younger age at surgery is the most consistently reported risk factor for the development of secondary glaucoma, the risk is expected to vary across the cohort. The use of surgical techniques may have differed across the duration of this study, and surgeries were performed by multiple surgeons. Especially, the posterior technique affecting the low‐dose group before 2017 at AUH differed substantially. However, due to our limited cohort size, we could only adjust for a limited number of variables. The high‐dose regimen was only given at RH until 2017 and could be adjusted based on surgeon preferences; however, it was markedly higher compared to the low‐dose group, allowing us to categorize the children in a low‐dose and high‐dose group. Additionally, we had no means of verifying whether the eye drops were given as prescribed. The main strength of this study is the population‐based approach, with inclusion of every child receiving cataract surgery in Denmark consecutively in a 12‐year period, as children were not operated elsewhere.

Further research is needed to validate our findings. Ideally, a randomized controlled trial with a larger cohort of children with AL <18 mm receiving low‐dose and high‐dose treatment could assess the effect on the development of glaucoma.

## 5 CONCLUSION

We report a lower risk of glaucoma 5 years after cataract surgery in children with axial lengths ≥18 mm receiving low‐dose versus high‐dose postoperative local and topical glucocorticoid treatment. This effect was not present in children with shorter axial lengths. We did not find any difference in the long‐term risk of treatment for visual axis opacification or pupillary membranes between the two treatment groups.

## FUNDING INFORMATION

The Danish Eye Research Foundation, Helsefonden, Fight for Sight, Synoptik Foundation, Fabrikant Einar Willumsens Mindelegat, Beckett‐Fonden, Th. Maigaards efterfølger fru Lily Benthine Lunds fond af 1.6.1978, Kong Christian den Tiendes Fond, and Helene and Viggo Bruuns Foundation. AL Solebo is supported by an NIHR Clinician Scientist grant (CS‐2018‐18‐ST2‐005). The sponsor or funding organization had no role in the design or conduct of this research.
